# Human Properdin Opsonizes Nanoparticles and Triggers a Potent Pro-inflammatory Response by Macrophages without Involving Complement Activation

**DOI:** 10.3389/fimmu.2018.00131

**Published:** 2018-02-12

**Authors:** Lubna Kouser, Basudev Paudyal, Anuvinder Kaur, Gudrun Stenbeck, Lucy A. Jones, Suhair M. Abozaid, Cordula M. Stover, Emmanuel Flahaut, Robert B. Sim, Uday Kishore

**Affiliations:** ^1^Biosciences, College of Health and Life Sciences, Brunel University London, Uxbridge, United Kingdom; ^2^Faculty of Science, Engineering and Computing, Kingston University, Kingston upon Thames, Surrey, United Kingdom; ^3^Department of Infection and Immunity, King Faisal Specialist Hospital and Research Centre, Riyadh, Saudi Arabia; ^4^Department of Infection, Immunity and Inflammation, University of Leicester, Leicester, United Kingdom; ^5^Université de Toulouse, CNRS, INPT, UPS, UMR CNRS-UPS-INP N°5085, 3 Paul Sabatier, Bât. CIRIMAT, Toulouse, France; ^6^Department of Biochemistry, University of Oxford, Oxford, United Kingdom

**Keywords:** carbon nanotubes, complement, properdin, thrombospondin repeats, phagocytosis, inflammation, cytokines

## Abstract

Development of nanoparticles as tissue-specific drug delivery platforms can be considerably influenced by the complement system because of their inherent pro-inflammatory and tumorigenic consequences. The complement activation pathways, and its recognition subcomponents, can modulate clearance of the nanoparticles and subsequent inflammatory response and thus alter the intended translational applications. Here, we report, for the first time, that human properdin, an upregulator of the complement alternative pathway, can opsonize functionalized carbon nanotubes (CNTs) *via* its thrombospondin type I repeat (TSR) 4 and 5. Binding of properdin and TSR4+5 is likely to involve charge pattern/polarity recognition of the CNT surface since both carboxymethyl cellulose-coated carbon nanotubes (CMC-CNT) and oxidized (Ox-CNT) bound these proteins well. Properdin enhanced the uptake of CMC-CNTs by a macrophage cell line, THP-1, mounting a robust pro-inflammatory immune response, as revealed by qRT-PCR, multiplex cytokine array, and NF-κB nuclear translocation analyses. Properdin can be locally synthesized by immune cells in an inflammatory microenvironment, and thus, its interaction with nanoparticles is of considerable importance. In addition, recombinant TSR4+5 coated on the CMC-CNTs inhibited complement consumption by CMC-CNTs, suggesting that nanoparticle decoration with TSR4+5, can be potentially used as a complement inhibitor in a number of pathological contexts arising due to exaggerated complement activation.

## Introduction

Nanoparticles including carbon nanotubes (CNTs) are considered attractive drug delivery platforms. However, their intended destination post-administration and associated cytotoxicity can be significantly altered by the innate immune system, which is likely to interact rapidly with the nanoparticles (1–3). A number of nanoparticles have been shown to activate a potent humoral wing of the innate immunity called the complement system (4–6). The complement system can influence the pharmacokinetics and biodistribution of the therapeutic nanoparticles since complement proteins are potent opsonins, acting as a bridge between nanoparticles and a range of innate and adaptive immune cells (2, 7). This interaction not only brings about clearance of nanoparticles by phagocytic cells (8) but also influences the inflammatory response (9).

**Figure 1:**
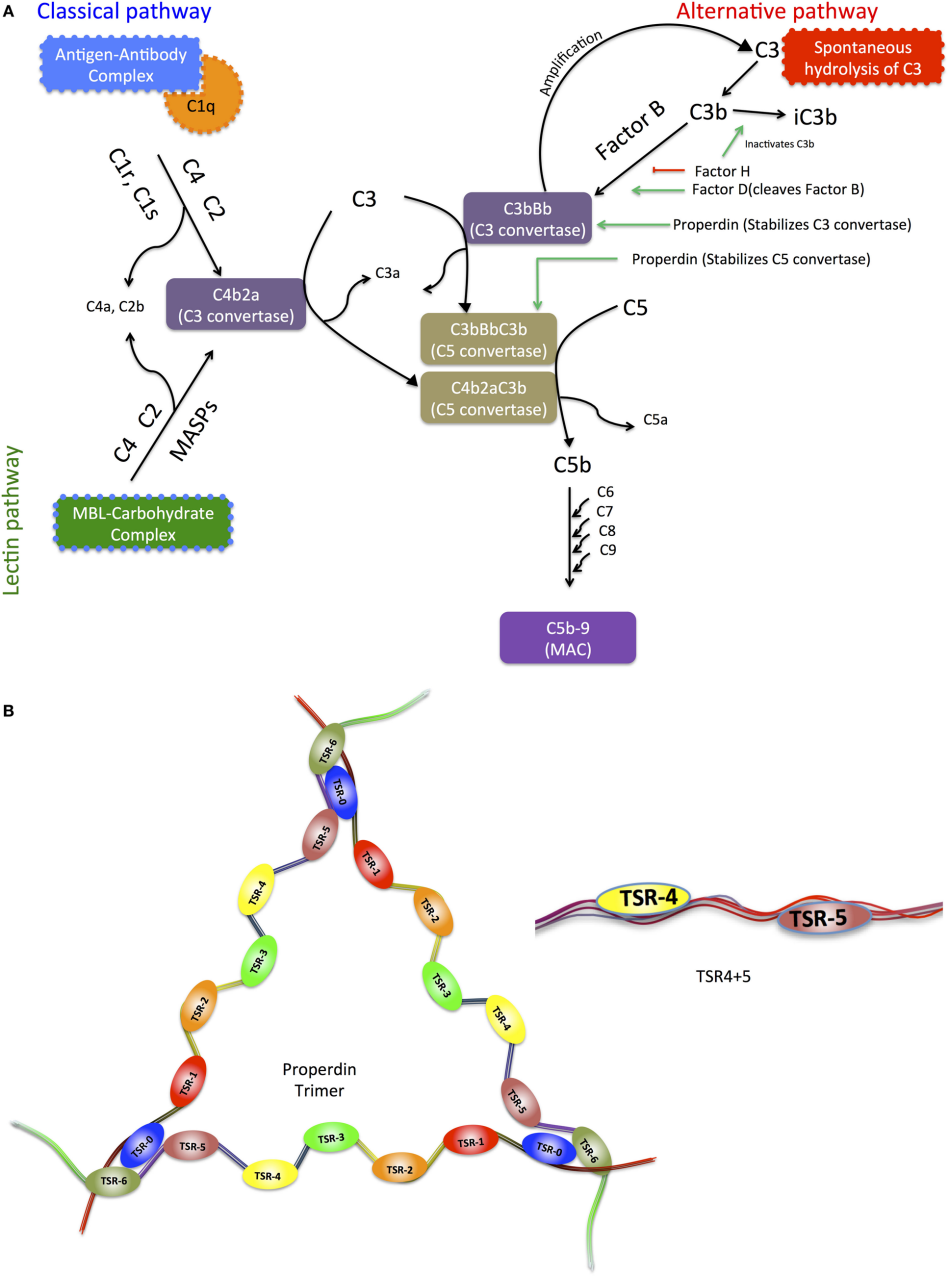
**(A)** Schematic diagram of the three pathways of the complement system, highlighting the upregulator (properdin) and downregulator (factor H) of the alternative pathway. The three complement systems, the classical, alternative, and lectin pathways merge in C5 convertase. C5 convertase cleaves C5 forming C5b, which combines with C6, C7, C8, and C9 to form the membrane attack complex (MAC). Activation of the classical pathway occurs upon recognition and binding of targets *via* C1q, which in turn activates C1r and C1s, and C1s cleaves C4 and C2. The lectin pathway is activated when mannose-binding lectin (MBL) or ficolins bind to microbial surfaces or other targets, and this binding activates MBL-associated serine proteases (MASPs), similar to C1s, which cleave C4 and C2. Classical and lectin pathway cleaved products of C4 and C2 form C3 convertase (C4bC2a), which cleaves C3 into C3b, the major opsonin of the complement system, binds covalently to targets. C3b also binds to C4b2a, altering its substrate preference to cleave C5 and thus forming C5 convertase C4b2a3b, which cleaves C5 into C5a and C5b. C5b assembles with C6, C7, C8, and C9 to form the MAC, which can cause disruption or lysis of target cells. The alternative pathway C3 convertase C3bBb is a homolog of C4b2a. It is formed by slow hydrolysis of C3 to form C3(H_2_O), which is similar to C3b in conformation. C3(H_2_O) forms a complex with factor B (FB), and FB in the complex is cleaved to form C3(H_2_O)Bb, plus Ba, by factor D (FD). C3(H_2_O)Bb itself cleaves C3 to form C3a plus C3b, and C3b then reacts with FB and FD to form C3bBb, in the same way as C3(H_2_O)Bb is formed. C3bBb is unstable, but is stabilized by properdin, which holds together the C345C domain in C3b and the vWF domain in Bb, and also repositions the thioester-containing domain in C3b that is involved in the decay process of C3 convertase. **(B)** Representative diagram of the modular organization of human properdin including thrombospondin repeats. Properdin is found in serum as monomers, dimers, trimers, and tetramers of a polypeptide made up of seven thrombospondin type I repeat (TSR) domains. It enhances alternative pathway activation by stabilization of C3 and C5 convertases. TSR0 is the *N*-terminal TSR. TSR4 and TSR5 modules expressed in tandem in E. coli have also been depicted.

The complement system consists of more than 40 plasma proteins circulating in the blood and tissue fluids, many as inactive zymogens, which upon sequential activation help to defend against infection and mount an immune response (10). Complement activation takes place *via* the classical, lectin, and alternative pathways (Figure 1A) (10–13). In the alternative complement pathway, properdin is an upregulator of complement activation.

Properdin interacts with the surface-bound ligands C3b, C3bB, or C3bBb. Once properdin binds both C3b and Bb, the unstable C3 convertase, which has a half-life of about 90 s is strongly stabilized, its half-life increasing by 5- to 10-fold (14). Thus, the intermediates C3bP (where P stands for properdin) and C3bBP generate C3bBbP on the target surfaces (14). This allows the generation of C3b in an amplification loop, resulting in deposition of many copies of C3b on a complement-activating surface. One C3b binds to C3bBb itself, forming C3bBbC3b, homologous to the classical pathway C5 convertase C4b2a3b, thus leading to C5 cleavage and lytic pathway (10).

The C3 activation cascade is downregulated on host cells by membrane-bound regulatory proteins: complement receptor 1 (CR1), membrane cofactor protein (MCP) and decay accelerating factor (DAF), soluble factor H and factor I. Factor H inhibits C3bBb formation by binding to C3b; C3b in the factor H–C3b complex is cleaved by factor I to iC3b. In addition, factor H enhances the decay of convertase activity by displacing Bb from the complex, thus inhibiting the activation of complement alternative pathway (15). CR1, factor H, and DAF bind to C3b and dissociate the Bb fragment, while factor H, CR1 and MCP are cofactors for the factor I-mediated cleavage of C3b to iC3b, thus preventing the amplification of activation of complement on the host cell surface. Complement facilitates the recognition by phagocytic cells of complement-activating particles *via* bound complement components, which are recognized by the receptors CR1, CR2, CR3, CR4, and CRIg. CR1, expressed on macrophages and neutrophils, binds C3b, leading to phagocytosis in the presence of immune mediators such as C5a (10, 16).

Properdin is found at a concentration of 25 µg/mL in plasma (17). Due to this low level, its local production by a variety of stimulated cells, such as neutrophils, endothelial cells, peripheral blood monocytes, dendritic cells, and T cells, may be important in localized activation of the alternative pathway (18). Properdin is made up of identical subunits of 53 kDa associating with each other in a head-to-tail manner (19, 20) to form cyclic polymers (dimers, trimers, and tetramers) dispersed in a ratio of 26:54:20 (21). The human properdin monomer has seven thrombospondin type I repeats (TSR; TSR0–TSR6). A typical TSR is 60 amino acids long and has sequence similarities with thrombospondin, circumsporozoite protein of malaria parasite, and a domain of complement component C9 (22, 23) (Figure 1B). TSR4 and TSR5 in properdin play an important role in binding to C3b and in the stabilization of C3 convertase (24–26).

Carbon nanotubes and their functionalized derivatives can activate complement *via* the classical and alternative pathways (4–6). Complement deposition can enhance their uptake by complement receptor-bearing macrophages and B cells, while downregulating the pro-inflammatory response that is otherwise induced by CNTs (8, 9). We have shown previously that C1q, the recognition subcomponent of the classical pathway, and factor H, the downregulator of the alternative pathway, can bind CNTs directly (8). The structural organization of properdin (multisubunit, multiple potential binding domains, and potential multivalency) appears to suggest a possible role as a soluble pattern recognition receptor (PRR). It has been shown that properdin can bind apoptotic T cells *via* sulfated glycosaminoglycan (GAG) and augment phagocytosis by macrophages (27). Furthermore, properdin can also bind to DNA exposed on apoptotic and necrotic cells in a C3b-independent manner (28). Recently, it has been shown that properdin locally produced by DCs and tolerogenic DCs can bind to bind to necrotic cells, confirming previous reports to implicate properdin as an independent recognition molecule. Furthermore, treatment of DCs with siRNA targeting properdin reduced the proliferation of allogenic T cells, and this effect was more pronounced when combined with IFN-γ stimulation. Interestingly, IFN-γ reduced the production of properdin and factor H in both types of DCs (29). This demonstrates that local production of properdin is crucial for the DC and T cell responses. Furthermore, the production of properdin by neutrophils (29, 30) was comparable to DCs, whereas factor H production was very low (29).

Macrophages of properdin-deficient mice have reduced M1 phenotype (IL-1β) and an increased production in the M2 phenotype (arginase-1, MCP-1, and IL-10) associated with tumour-promoting activity. This suggests that the deficiency in properdin can modulate macrophages toward an M2 phenotype, which enhances the tumor environment (31).

Properdin binds to surfaces of several pathogens such as *Neisseria gonorrhoeae* (32), *Salmonella typhimurium* lipopolysaccharide (LPS), *Neisseria meningitidis* lipooligosaccharide (33), and *Chlamydia pneumoniae* (34), which leads to complement activation (15). In addition, recombinant properdin enhanced the opsonization of *N. meningitidis* and *S. pneumonia* by human serum *in vitro* (16). Properdin also binds to Zymosan, *Escherichia coli* (E. coli) strains, live human leukemia T cell lines, and rabbit erythrocytes, suggesting that properdin binding to these surfaces demonstrates its role as a PRR, initiating alternative pathway on target surfaces.

Properdin binds to NKp46 expressed on NK cells and innate lymphoid cell (ILC)1 and ILC3. The control of infection by properdin was dependent on NKp46 and group 1 ILCs. The control of meningococcal infection was not dependent on membrane attack complex, further confirming the pattern recognition role of properdin (35).

Here, we show that properdin acts as a pattern recognition receptor, binds to CNTs, *via* at least domains TSR4+5, and enhances their phagocytosis by macrophages (opsonizes), in addition to promoting a robust pro-inflammatory immune response. The main C3b-binding domains of properdin, TSR4 and TSR5, when coated together on CNTs, acted as a potent inhibitor of complement alternative pathway activation, raising the possibility of regulating inflammatory response and complement activation on synthetic material surfaces.

## Materials and Methods

### Expression and Purification of Recombinant Human Properdin in Human Embryonic Kidney (HEK) Cells

Full-length human properdin gene was subcloned as an EcoRI-XbaI cassette into pSecTag-C (Life Technologies), using terminal primers 5′-GAATTCGACCCCGTGCTCTGCTTCAC-3′ and 5′-TCTAGAGAGTTCCTCTTCC TCAGGGTCTTTG-3′, yielding the construct pLK-FL. The cDNA clone of human properdin was used as a template in the PCR reaction that was originally isolated from U937 cells stimulated by PMA and subcloned in the pBluescript vector (19, 24). HEK cells were plated in 24-well plates at a concentration of 0.5–1.25 × 10^5^ cells per well in 0.5 mL of complete DMEM-F12 medium [10% v/v fetal calf serum (FCS), 2 mM l-glutamine, 100 U/mL penicillin, and 100 µg/mL streptomycin] and left at 37°C, 5% CO_2_ in humidified atmosphere. Once the cells reached about 80% confluence, the growth medium was replaced with fresh 0.5 mL/well of complete DMEM-F12 medium. Up to 5 µg of pLK-FL DNA in 100 µL of Opti-MEM^®^ I Medium (Gibco) without serum was added to each well for transfection. Up to 1.75 µL of Lipofectamine LTX^®^ Reagent (Invitrogen) was added into the above-diluted Opti-MEM^®^-DNA solution, mixed gently, and incubated for 30 min at room temperature to form DNA-Lipofectamine LTX^®^ Reagent complexes, 100 µL of which was added directly to each well containing HEK cells, swirled gently, and incubated at 37°C in CO_2_ incubator for up to 24 h before examining for protein expression and secretion. Supernatant was collected and tested for the presence of properdin by direct ELISA.

Recombinant full-length properdin was purified by passing the supernatant through an antiproperdin IgG (monoclonal) affinity column, kindly provided by Dr. C. Koch, State Serum Institute, Copenhagen, Denmark. The column (anti-Properdin-Sepharose) was washed with three bed volumes of HEPES buffer (10 mM HEPES, 140 mM NaCl, 0.5 mM EDTA, pH 7.4). Bound properdin was eluted in 1 mL fractions using 3 M MgCl_2_. The peak fractions were dialyzed against HEPES buffer overnight at 4°C. Minor contaminants were further removed by applying the dialyzate on to a DEAE Sepharose ion exchange column (GE Healthcare), which was first equilibrated with three bed volumes of Tris buffer (50 mM Tris–HCl, pH 7.5, 50 mM NaCl, 5 mM EDTA). Properdin did not bind to the DEAE Sepharose and was recovered in the flow-through, whereas other contaminants remained bound to the resin. SDS-PAGE analysis was carried out to confirm the purity of recombinant properdin.

### Expression and Purification of Recombinant Human TSR4+5 Modules in Tandem, Fused to Maltose-Binding Protein *in E. coli*

Recombinant human TSR4+5 fused with maltose-binding protein (MBP) was expressed using *E. coli* BL21 strain (Life Technologies) (26). Overnight primary bacterial cultures were grown in Luria-Bertani medium with 100 µg/mL ampicillin with shaking (200 rpm) at 37°C until an A_600_ of 0.6–0.8 was reached. Expression was then induced with 0.4 mM isopropyl β-d-thiogalactoside (Sigma) and continued for 3 h at 37°C. The cell pellet, recovered after centrifugation at 4,500 rpm for 10 min, was resuspended in lysis buffer (20 mM Tris–HCl, pH 8.0, 0.5 M NaCl, 1 mM EDTA, 0.25% v/v Tween 20, 5% v/v glycerol) containing 100 µg/mL lysozyme (Sigma) and 0.1 mM phenylmethanesulfonyl fluoride (Sigma) and incubated for 1 h at 4°C on a rotary shaker. The lysate was then sonicated (using a Soniprep 150; MSE, London, UK) at 60 Hz for 30 s with an interval of 2 min on ice (12 cycles). The sonicate was centrifuged at 10,000 *g* for 15 min at 4°C. The supernatant was collected and diluted 5-fold with buffer I (20 mM Tris–HCl, pH 8.0, 100 mM NaCl, 1 mM EDTA, 0.25% v/v Tween 20) and passed through an amylose resin column (New England Biolabs), previously equilibrated in buffer I. The affinity column was washed with buffer I without Tween 20 and with 1 M NaCl, followed by buffer II (20 mM Tris–HCl, pH 8.0, 100 mM NaCl, 1 mM EDTA). The TSR4+5 fusion protein was then eluted with buffer II containing 10 mM maltose (Sigma). Minor contaminants were further removed by applying the fusion protein to a Q-Sepharose column (Sigma) equilibrated with three column volumes of low-salt buffer (50 mM Tris–HCl, pH 7.5, 100 mM NaCl, 5 mM EDTA, pH 7.5). After extensive washing with low-salt buffer, the fusion protein was step eluted with 200 mM NaCl. The peak fractions were then passed through Pierce™ High Capacity Endotoxin Removal Resin to remove LPS. Endotoxin levels in the purified protein samples were analyzed using the QCL-1000 Limulus amebocyte lysate system (Lonza). The assay was linear over a range of 0.1–1.0 EU/mL (10 EU = 1 ng of endotoxin), and the endotoxin levels were <5 pg/µg of the recombinant proteins. MBP protein used as a control was expressed in *E. coli* using pMal-c vector (New England Biolab) and purified, as described above for MBP-TSR4+5.

### Preparation and Transmission Electron Microscopy (TEM) of Carboxymethyl Cellulose-Coated Carbon Nanotubes (CMC-CNTs)

The synthesis and characterization of double-walled carbon nanotubes (DWNTs) has been described earlier (36, 37). After catalyst elimination by non-oxidizing HCl treatment, the DWNTs were washed with deionized water. For functionalization, wet DWNTs (corresponding to a dry amount of 100 mg) were added to a solution of 100 mg of carboxymethyl cellulose (CMC; Sigma 21901) in PBS. After mixing, the suspension was freeze dried to obtain a 50:50 homogeneous dry mixture of DWNTs and CMC. The samples were resuspended in PBS + 5 mM EDTA pH 7.4, and then centrifuged at 8,000 *g* for 5 min for the removal of aggregates. After centrifugation, the non-sedimented CNTs were washed to remove excess CMC by vacuum filtration using 0.2 µm Whatman polycarbonate filter with PBS-EDTA, pH 7.4. Functionalized CNTs (CMC-CNTs) were then resuspended in PBS-EDTA. For oxidized DWNTs, 100 mg of DWNTs were added to 100 mL of HNO_3_ (3 M) and placed in ultrasonic bath for 30 min and refluxed at 130°C for 24 h. The solution was left to cool at room temperature. The solution was washed and filtered on a polypropylene membrane (0.45 μm). For electron microscopy, 2 µL of well-dispersed CMC-CNTs were adsorbed onto carbon-coated grids. Micrographs were recorded using a JEOL 2100 FEG-TEM operating at 80 Kv, and the images were processed using Gatan microscopy suite software (Gatan, Inc.). Surface visualization of CMC-CNTs was performed using a Zeiss Supra 35vP scanning electron microscope operating at 5 kev.

### Coating of CMC-CNTs and Ox-CNTs with Properdin and TSR4+5

Purified properdin, TSR4+5, MBP, or BSA (Bovine serum albumin) were incubated in a w/w ratio of 2:1 with 100 µg of CMC-CNTs or Ox-CNTs in the affinity buffer (50 mM Tris–HCl, pH 7.5, 150 mM NaCl, 5 mM CaCl_2_), overnight at 4°C. Excess protein was removed by repeated centrifugation and washing at 17,000 *g* for 10 min to wash away any unbound proteins trapped within the CNTs. CNTs were redispersed in affinity buffer between centrifugations.

### Western Blotting to Detect Protein Binding to CNTs

Carbon nanotubes with bound protein were run on SDS-PAGE under reduced conditions, and protein bands were transferred on to a nitrocellulose membrane in transfer buffer (25 mM Tris, 192 mM glycine, 20% v/v methanol, pH 8.3) at 320 mA for 2 h. The membrane was then blocked with 5% semi-skimmed milk powder (Tesco, UK) in PBS, pH 7.4 (Sigma) overnight at 4°C. Rabbit anti-human properdin (0.92 mg IgG/mL) polyclonal antibodies were diluted 1:500 in PBS and incubated with the membrane for 2 h at room temperature. The membrane was washed three times, 10 min each, with PBS + 0.05% Tween 20 (PBST). Protein A–horseradish peroxidase (1:1,000; Thermo Scientific) in PBS was added and left at room temperature for 1 h. The blot was washed again with PBST three times, and the color was developed using 3,3′-diaminobenzidine (DAB) (Sigma-Aldrich).

### Complement Consumption Assays

To measure complement consumption in human serum *via* the alternative pathway, properdin, TSR4+5, or MBP were precoated on CMC-CNTs in 1:1 w/w ratio in affinity buffer overnight at 4°C, followed by washing, as described above to remove unbound protein. Protein-coated CMC-CNTs and zymosan (positive control) were incubated with human serum (1/5 dilution) in DGVB-Mg-EGTA buffer (2.5 mM sodium barbital, 71 mM NaCl, 7 mM MgCl_2_, 10 mM EGTA, 2.5% w/v glucose, 0.1% gelatin, pH 7.4) for 1 h at 37°C. Serum diluted 1/5 and incubated with no additions was the negative control. After incubation, CNTs were removed by centrifugation at 17,000 *g* for 10 min, and the ability of the supernatant to lyse rabbit erythrocytes (TCS Biosciences) was tested. Rabbit erythrocytes were washed by repeated centrifugation for 10 min, 700 g in PBS + 5 mM EDTA, pH 7.4 until the supernatant was clear. The cell concentration was adjusted to 1 × 10^9^/mL in DGVB-Mg-EGTA. Then, 100 µL of these rabbit erythrocytes was added to 100 µL of serum supernatant samples or to undiluted normal human serum and incubated for 1 h at 37°C. After incubation, cells were centrifuged (700 *g*, 10 min) and released hemoglobin in the supernatant was measured at 541 nm. Total hemolysis (100%) was measured by lysing rabbit erythrocytes with undiluted normal human serum. Background spontaneous hemolysis was determined by incubating rabbit erythrocytes with DGVB-Mg-EGTA buffer. Percentage complement consumption was calculated using (*C* − Ci)/*C* × 100%, where *C* represents the % hemolysis of the negative control, and Ci is the % hemolysis with the CMC-CNT- or zymosan-treated sample.

To check whether the CMC-CNTs with bound properdin retained capacity to activate the complement alternative pathway, we used properdin-deficient serum obtained from the properdin gene-deficient mice (38). Genotyped mice were bled under terminal anesthesia. Blood was spun, and serum was transferred to a tube and stored at −20°. Properdin-coated CMC-CNTs (Properdin-CNT), TSR-coated CMC-CNTs (TSR4+5-CNT), and CMC-CNT alone were incubated with properdin-deficient serum (1/2 dilution) in DGVB-Mg-EGTA buffer and incubated for 1 h at 37°C. After incubation, CNTs were removed by centrifugation at 17,000 *g* for 10 min, and serum was collected. To each of the collected sera, purified properdin (1 µg/mL) was added, and the reconstituted sera were tested for the lysis of rabbit erythrocytes, as described above.

### Biotinylation of CMC-CNTs

Carboxymethyl cellulose-coated carbon nanotubes were biotinylated as follows: 1 mg of CMC-CNTs was suspended in 1 mL 0.1 M MES buffer [2-(*N*-morpholino) ethanesulfonic acid, pH 5]. 1 mg of pentylamine biotin (Pierce, Thermo Fisher Scientific) in the presence of 4 µg EDC [1-Ethyl-3-(3-dimethylaminopropyl) carbodiimide] was added to the CMC-CNT suspension and stirred for 2 h at room temperature. The reaction was stopped by adding 50 µL of 0.1 M ethanolamine (Sigma). The resulting biotin-CMC-CNTs were dialyzed extensively against PBS (pH 7.4) to remove remaining reactants and MES.

### Uptake of CMC-CNT into THP-1 Cells, Observed by Fluorescence Microscopy

Uptake of biotinylated CMC-CNTs, coated with properdin or MBP-TSR4+5, was examined using differentiated THP-1 macrophages. For fluorescence microscopy, 1 × 10^5^ THP-1 cells were plated on 13-mm coverslips and treated for 24 h with 100 nM Phorbol myristate acetate (PMA; Sigma) in complete RPMI 1640 containing 10% v/v FCS, 2 mM l-glutamine, 100 U/mL penicillin, 100 µg/mL streptomycin, and 1 mM sodium pyruvate. Differentiated THP-1 cells were washed three times with PBS to remove excess PMA and then rested for 24 h in complete RPMI 1640 as above prior to exposure to the CNTs. Cells were washed three times with PBS and were exposed to 4 µg/mL biotinylated CMC-CNT coated with properdin (Properdin-CNTs), MBP-TSR4+5 (TSR4+5-CNT), or biotin-CMC-CNTs alone in 500 µL of serum-free RPMI 1640 medium for 2 h. Cells were washed twice with PBS, fixed with 4% paraformaldehyde for 10 min, washed, and processed for fluorescence microscopy. The cells on coverslips were permeabilized using permeabilizing buffer (20 mM HEPES, pH 7.4, 300 mM sucrose, 50 mM sodium chloride, 3 mM MgCl_2_, 0.5% Triton X-100) for 5 min on ice. The cells were then stained for 30 min with 1.6 µM Hoechst 33342 (Life Technology), 2 µg/mL Alexa-Fluor546-conjugated wheat germ agglutinin (Invitrogen) and Alexa fluor 488-conjugated streptavidin (Thermo Scientific) to reveal biotinylated CMC-CNTs. Cells were washed twice, mounted using Citifluor anti-fade (Citifluor, UK), and observed under a Nikon Eclipse TE2000-S confocal microscope with 62 X oil lens.

To observe nuclear translocation of NF-κB, permeabilized cells were incubated with rabbit anti-NF-κB p65 polyclonal antibodies (Santa Cruz Biotech), followed by secondary Alexa Fluor 488-goat anti rabbit antibody, and observed with Leica Fluorescent microscope using LAS software (Leica Microsystems).

For quantification, 5 × 10^5^ THP-1 cells were plated in 12-well plates and differentiated with PMA for 24 h and rested for 24 h in complete RPMI media. Cells were washed with PBS and exposed with 4 µg/mL biotinylated CMC-CNT coated with properdin (Properdin-CNTs), MBP-TSR4+5 (TSR4+5-CNT), or biotin-CMC-CNTs alone in 500 µL of serum-free RPMI 1640 medium for 2 h. Cells were washed three times with PBS and lysed with lysis buffer (10 mM HEPES, 20 mM NaCl, 0.5 mM EDTA, 1% v/v Triton X 100). An ELISA type assay was employed to quantify the amount of CNTs taken up by THP-1 cells (6). Microtiter wells (NUNC, polysorb) were coated with 100 µL Avidin (Pierce) at 50 µg/mL in 0.1 M carbonate bicarbonate buffer, pH 9 (Sigma) for 1 h at RT, followed by blocking with 0.05% of BSA for 1 h at RT. 50 µL of a solution or cell lysate containing biotin-CMC-CNTs and 50 µL of 0.05% BSA were added in each well and incubated for 1 h at RT. The plate was washed with PBS to remove unbound CNTs, and then incubated with 1:2,000 dilution of Streptavidin-HRP (Sigma) for 1 h at RT. Following washing again, O-phenylenediamine dihydrochloride (Sigma) was used as a substrate for the HRP, and the yellow 2, 3-diaminophenazine product was read at 450 nm.

### Measurement of THP-1 Cell Cytokine and Transcription Factor mRNA Expression Using Quantitative RT-PCR

In a 12-well cell culture plate (Nunc), THP-1 cells (1 × 10^6^/well) were differentiated with 100 nM PMA in RPMI 1640 complete medium for 24 h and then rested (without PMA) for 24 h. Cells were washed three times with PBS prior to the addition of 10 µg/mL of properdin-CMC-CNT, MBP-TSR4+5-CMC-CNT, or CMC-CNT alone to wells in serum-free RPMI 1640 medium and incubated for 30, 60, 120, or 360 min. Cells at each time point were washed with PBS and lysed within the wells using lysis buffer from GenElute Mammalian Total RNA Purification Kit (Sigma-Aldrich). Total RNA was extracted from the lysate using the GenElute Mammalian Total RNA Purification Kit (Sigma-Aldrich). To inactivate both DNase I and RNase, samples were heated at 70°C for 10 min and subsequently chilled on ice. A NanoDrop 2000/2000c spectrophotometer (Thermo-Fisher Scientific) was used to determine the amount and purity (260/280 nm ratio) of RNA. The cDNA was synthesized using High Capacity RNA to cDNA Kit (Applied Biosystems). Primers (Table 1) were designed using Primer-BLAST.^1^

**Table 1:** Terminal primers used for qPCR analysis.

Targets	Forward primer	Reverse primer
18S	ATGGCCGTTCTTAGTTGGTG	CGCTGAGCCAGTCAGTGTAG
IL-1β	GGACAAGCTGAGGAAGATGC	TCGTTATCCCATGTGTCGAA
IL-6	GAAAGCAGCAAAGAGGCACT	TTTCACCAGGCAAGTCTCCT
IL-10	TTACCTGGAGGAGGTGATGC	GGCCTTGCTCTTGTTTTCAC
IL-12	AACTTGCAGCTGAAGCCATT	GACCTGAACGCAGAATGTCA
TGF-β	GTACCTGAACCCGTGTTGCT	GTATCGCCAGGAATTGTTGC
TNF-α	AGCCCATGTTGTAGCAAACC	TGAGGTACAGGCCCTCTGAT
NF-κB	GTATTTCAACCACAGATGGCACT	AACCTTTGCTGGTCCCACAT
NLRP3	GCCATTCCCTGACCAGACTC	GCAGGTAAAGGTGCGTGAGA

The qPCR reaction mixture included 5 µL Power SYBR Green MasterMix (Applied Biosystems), 75 nM forward and reverse primers and 500 ng template cDNA in a 10 µL reaction volume. PCR was performed using a Step One Plus Real-Time PCR System (Applied Biosystems). Human 18S rRNA target was used as an endogenous control. Data were analyzed using the Step One software v2.3 (Applied Biosystems). Ct (cycle threshold) values for each cytokine target gene were calculated. Relative expression of each cytokine target gene was calculated using the relative quantification (RQ) value, using the equation RQ = 2^−ΔΔCt^ for each cytokine target gene and comparing relative expression with that of the 18S rRNA constitutive gene product. Assays were conducted in triplicate.

### Multiplex Cytokine Array Analysis

Supernatant from THP-1 cells, incubated with non-biotinylated CMC-CNT, properdin-CMC-CNT, and MBP-TSR4+5-CMC-CNT for 24 and 48 h, were collected for measuring secreted cytokines, chemokines, growth factors, and other ligands and receptors. The analytes were measured using MagPix Milliplex kit (EMD Millipore) following the manufacturer’s protocol. 25 µL of assay buffer was added to each well of a 96-well plate, followed by addition of 25 µL of standard, control, or supernatant of THP-1 cells. 70 µL of a mixture of 36 individual capture magnetic beads was added to 3.5 mL of diluent buffer, vortexed, and 25 µL of the magnetic beads coupled to analytes were added to each well containing assay buffer, samples, and controls and incubated for 18 h at 4°C. The plate was washed with assay buffer, and 25 µL of detection antibodies (EMD Millipore) were incubated with the beads for 1 h at room temperature. 25 µL of streptavidin–phycoerythrin was then added to each well and incubated for 30 min at room temperature. Following the washing step, 150 µL of sheath fluid was added to each well, and the plate was read using the Luminex Magpix instrument. Assays were carried out in duplicate.

### Statistical Analysis

Statistical analysis was conducted using GraphPad Prism version 7.0 (GraphPad Software). An unpaired two-sided *t*-test and multiple *t*-test using Holm–Sidak method was used on the data for any significant difference between uncoated and protein coated CNTs. *P* values were computed and graphs compiled and analyzed.

## Results

### Recombinant Full-Length Properdin and MBP-TSR4+5 Bind CMC-CNTs

**Figure 2:**
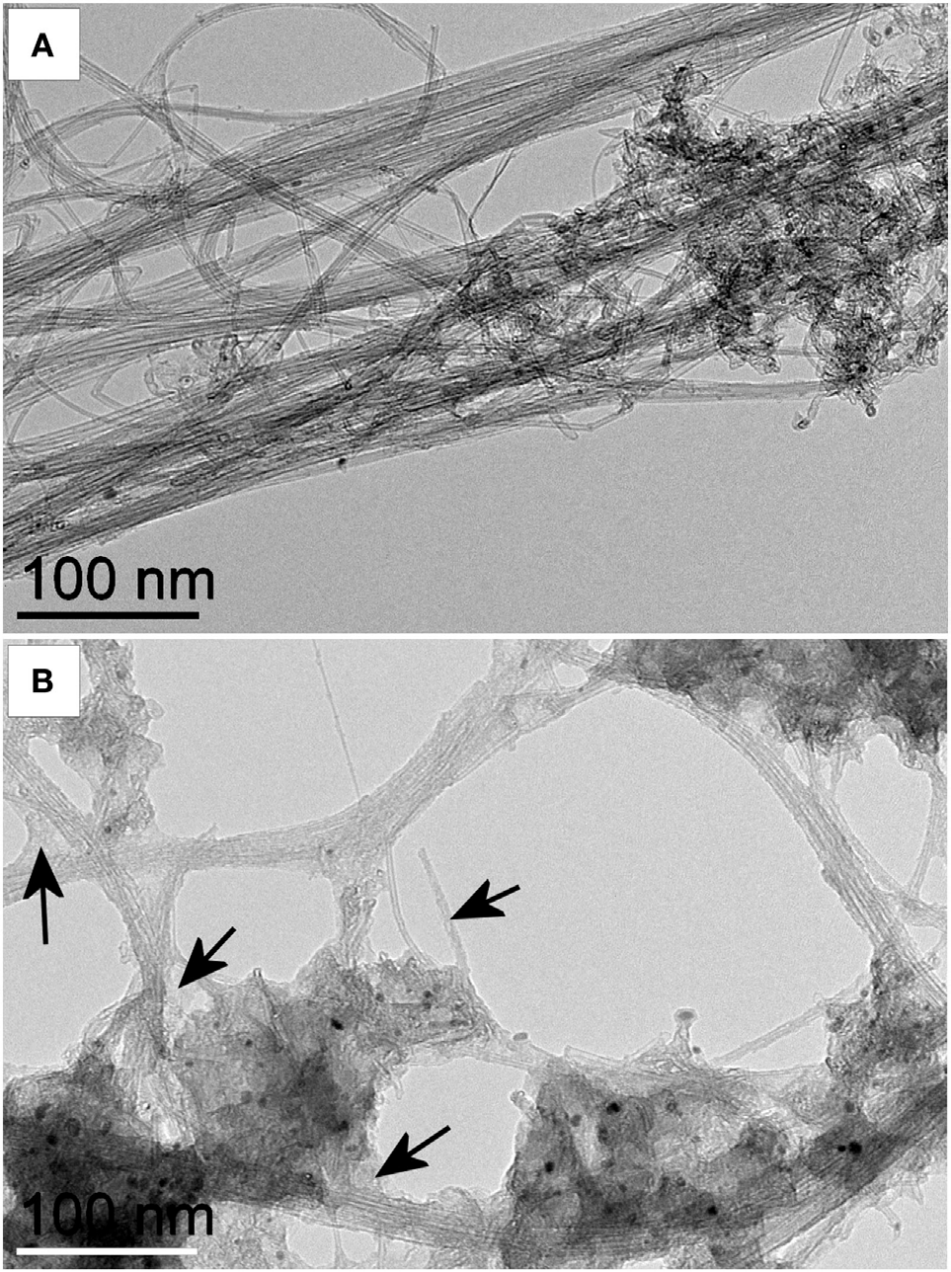
Transmission electron microscopy (TEM) images of pristine and carboxymethyl cellulose-coated carbon nanotubes (CMC-CNTs). High-resolution TEM image of **(A)** pristine DWCNTs and **(B)** CMC-coated DWCNTs. Images were acquired at the same magnification. Black arrows indicate examples of places where the carboxymethyl cellulose (CMC) coating on the nanotubes is easily visible. In general, after CMC coating, the image seems blurred and shows much less details. Double-walled carbon nanotubes were coated with CMC and dispersed in water. Suspended CMC-CNTs were adsorbed on a carbon grid. The images were recorded with a Jeol 2100 transmission electron microscope operating at 80 kV. Scale bar is 100 nm.

Functionalized DWNTs were well dispersed in aqueous solution. High-resolution TEM images of pristine DWCNTs (Figure 2A) and CMC-coated DWCNTs (Figure 2B) revealed the places where the CMC coating on the nanotubes was easily visible. In general, following CMC coating, the image seems blurred and shows much less details.

**Figure 3:**
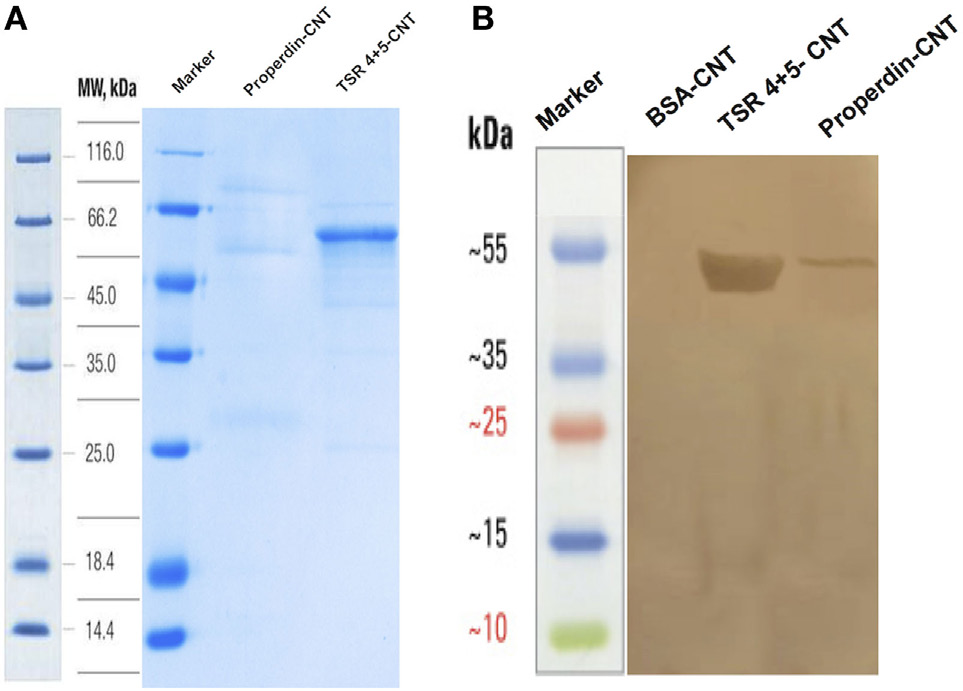
Carboxymethyl cellulose-coated carbon nanotubes (CMC-CNTs) stably bind to purified human properdin and recombinant TSR4+5, as shown *via* SDS-PAGE **(A)** and western blot **(B)**. **(A)** CMC-CNTs were incubated with recombinant human properdin, or thrombospondin type I repeat (TSR) 4 + 5-MBP fusion protein and BSA overnight in the affinity buffer. Carbon nanotubes (CNTs) were washed extensively *via* centrifugation and run on an SDS-PAGE (12%) under reduced conditions. Properdin migrated at ~55 kDa and MBP-TSR4+5 migrated as a single band at ~53 kDa. **(B)** In parallel, the SDS-PAGE was transferred onto nitrocellulose membrane for 2 h at 320 mA. The blot was probed with anti-human properdin (polyclonal) antibodies, followed by protein A–HRP conjugate and developed using 3,3′-diaminobenzidine.

We analyzed the binding of purified properdin as well as TSR4+5 modules (Figure 3A) to CNTs after preincubation of the proteins with CMC-CNTs followed by washing with PBS extensively *via* centrifugation. Western blot analysis revealed that properdin and MBP-TSR4+5 bound CMC-CNTs and appeared in the 12% SDS-PAGE at their expected molecular weight at ~55kDa (Figure 3B). CNTs remained in the loading wells. Furthermore, properdin and TSR4+5 also bound to Ox-CNTs efficiently (data not shown). This suggested that the properdin and TSR4+5 interaction with CNTs is likely to be through charge pattern/polarity recognition of the CNT surface and not due to CMC.

### MBP-TSR4+5 Coated on CMC-CNTs Inhibited Complement Consumption *via* the Alternative Pathway

**Figure 4:**
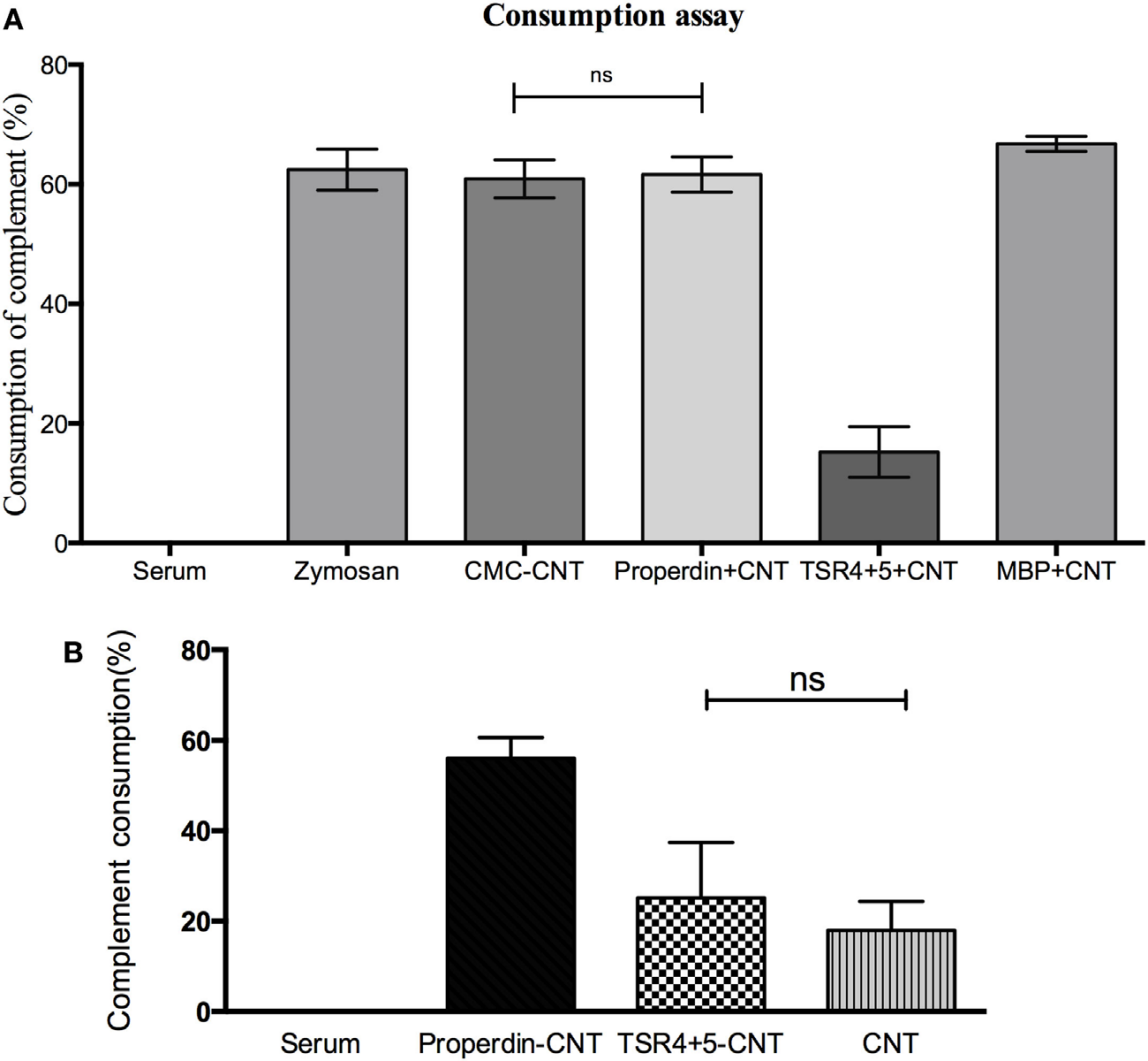
**(A)** Carboxymethyl cellulose-coated carbon nanotube (CMC-CNT) bound properdin activates the alternative pathway, while thrombospondin type I repeat (TSR)4 + 5-coated carbon nanotubes (CNTs) fail to consume complement: Properdin, TSR4+5, or maltose-binding protein (MBP)-coated CMC-CNTs were incubated with human serum (1/5 dilution in DGVB-Mg-EGTA buffer) for 1 h at 37°C. Samples were spun down, and serum was collected for consumption assay. Zymosan without CNTs was used as a positive control, and serum alone without CNTs was used as a negative control (zero consumption). **(B)** Only properdin-coated CNTs allow complement consumption in the presence of properdin-deficient serum derived from properdin gene knockout mice. Protein-coated CMC-CNTs were incubated with properdin-deficient serum diluted (1:2) with DGVB-Mg-EGTA buffer for 1 h at 37°C. The samples were centrifuged and properdin-deficient serum supernatant was collected. Properdin was added to the serum to give a final concentration of 1 µg/mL. Serum with reconstituted properdin was assayed for complement consumption. The experiments were repeated three times; error bars represent ± SD. A two-tailed, unpaired *t*-test was performed on the data to determine significant differences in complement consumption of properdin coated CNTs and TSR4+5-coated CNTs with CNTs only. All these comparisons were significant (*P* < 0.05), except where shown [not significant (ns), *P* > 0.05].

Properdin-coated and MBP-TSR4+5-coated CMC-CNTs were assessed for their ability to activate the complement alternative pathway (Figure 4A) compared with uncoated CMC-CNTs. Zymosan was used as a positive control that consumed complement. Properdin coating of CMC-CNT did not interfere with the alternative pathway activation; it allowed complement consumption by the CNTs to the same extent as CMC-CNT alone. MBP also did not diminish consumption. However, TSR4+5-coated CMC-CNTs showed ~60% less complement consumption, suggesting that the CNT-surface bound TSR4+5 acted as an inhibitor of the complement alternative pathway, similar to its inhibitory properties in solution (26). These results suggest that properdin is likely to bind to CNTs *via* TSR4+5 and that precoated TSR4+5 can inhibit the binding of properdin (from the serum), and thereby diminish alternative pathway activation. Properdin-coated CMC-CNTs also consumed complement (activated complement) *via* the alternative pathway (Figure 4B) when properdin-deficient serum was used. Complement consumption was increased by ~60% compared to uncoated CMC-CNTs. TSR4+5-coated CNTs were not significantly different in consumption from uncoated. This suggested that the CNT-bound properdin still retained its activity of promoting alternative pathway activation.

Figure 4 suggests that properdin binding is a dominant factor in alternative pathway activation by CMC-CNT. In Figure 4A (using normal human serum), it is shown that CMC-CNT, P-CMC-CNT, and MBP-CMC-CNT all consume about 60% of the (alternative pathway) complement activity in the serum, whereas TSR4+5-CMC-CNT consume only about 15%. In Figure 4B (properdin-deficient mouse serum), only P-CMC-CNT allow extensive consumption: CMC-CNT and TSR4+5 CMC-CNT show much lower consumption. Therefore, properdin (on the P-CMC-CNT) has a very large effect.

### Properdin, but Not TSR4+5, Enhanced CMC-CNT Uptake by THP-1 Cells

**Figure 5:**
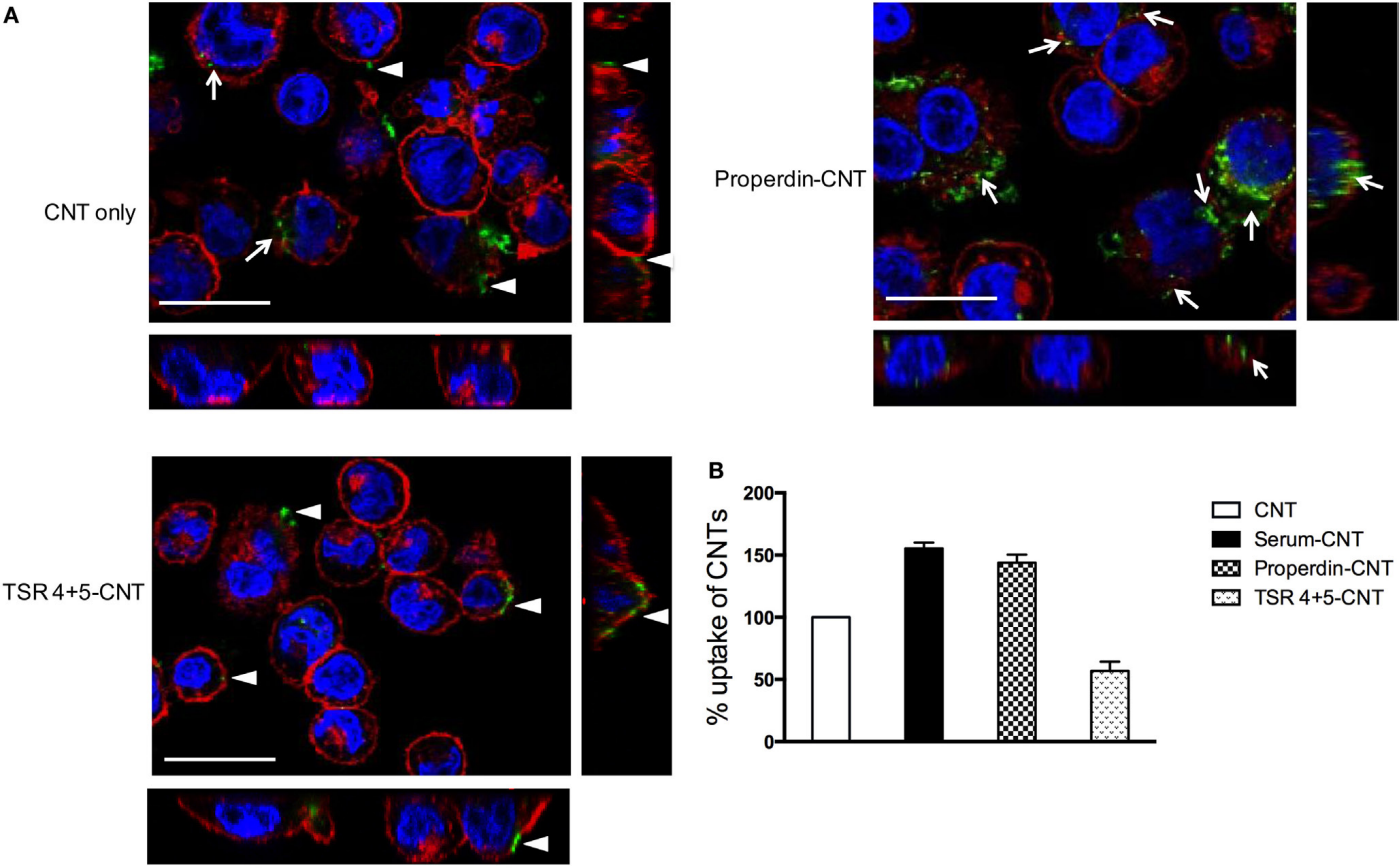
**(A)** Properdin-coated carboxymethyl cellulose-coated carbon nanotube (CMC-CNT) show enhanced uptake by THP-1 cells. To observe internalization of carbon nanotubes (CNTs), PMA-differentiated THP-1 cells were treated with properdin or MBP-TSR4+5-coated biotinylated CMC-CNTs and uncoated biotinylated CMC-CNTs for 2 h. Cells were washed, fixed, permeabilized, and stained with Alexafluor-488-labeled Streptavidin (green) to reveal internalized biotin-CMC-CNTs (arrows). Alexafluor546-conjugated wheat germ agglutinin was used to reveal plasma membrane (red), and the nucleus was stained with Hoechst 33342 (Blue). Images shown are single sections (including side images) taken with a Nikon confocal microscope; scale bar, 20 µm arrow heads point to the CNTs adhering to the plasma membrane. **(B)** TSR4+5-coated CNTs show reduced uptake by THP-1 cells. To quantify the amount of uptake of protein coated or uncoated CMC-CNTs, THP-1 cells were incubated with 4 µg/mL of protein-coated biotin CMC-CNTs or uncoated CMC-CNTs for 2 h. The cells were lysed, and the amount of CNTs was quantified by an ELISA type assay. All experiments were done in triplicate; error bars represent mean ± SEM. A two-tailed, unpaired *t*-test was performed on the data to determine significant differences in uptake of CNTs between properdin-coated CNTs and TSR4+5-coated CNTs with CNTs only. All these comparisons were significant (*P* < 0.05), except where shown [not significant (ns), *P* > 0.05].

Although properdin and TSR4+5 bound to nanoparticles, only full-length properdin, and not TSR4+5, was able to enhance uptake of CNTs by differentiated THP-1 cells at 2 h (Figure 5) in the absence of added complement (serum). Previous studies have reported an enhanced phagocytosis of nanoparticles in a complement-dependent and complement-independent manner. Precoating with C1q enhanced uptake of CNTs by U937 monocytic cells and human monocytes, whereas factor H, a negative regulator of the complement system, did not (8). As shown in Figure 5, Alexa Fluor 488-conjugated streptavidin-labeled biotin-CMC-CNT (green) did not show a significant level of uptake at 2 h. Properdin-CMC-CNT showed considerably enhanced uptake by THP-1 cells, compared to CMC-CNT alone and TSR4+5-CMC-CNT. The CMC-CNTs (green) were observed within the cell membrane stained with Alexa Fluor 546-conjugated wheat germ agglutinin (red) and the nucleus (Hoechst, blue). The qualitative confocal sections (right panel) with higher magnification revealed properdin-CMC-CNT within the cell; however, very few CMC-CNTs could be seen intracellularly in the case of TSR4+5-CMC-CNT (Figure 5), suggesting that an intact properdin molecule is required for this function as an “opsonin.” This also indicates that interaction of properdin with macrophage may not be limited to TSR4+5 only, and other TSR modules may be involved.

### Pro-inflammatory Cytokines Are Upregulated by Properdin-Coated CMC-CNTs, as Revealed by qPCR Analysis

**Figure 6:**
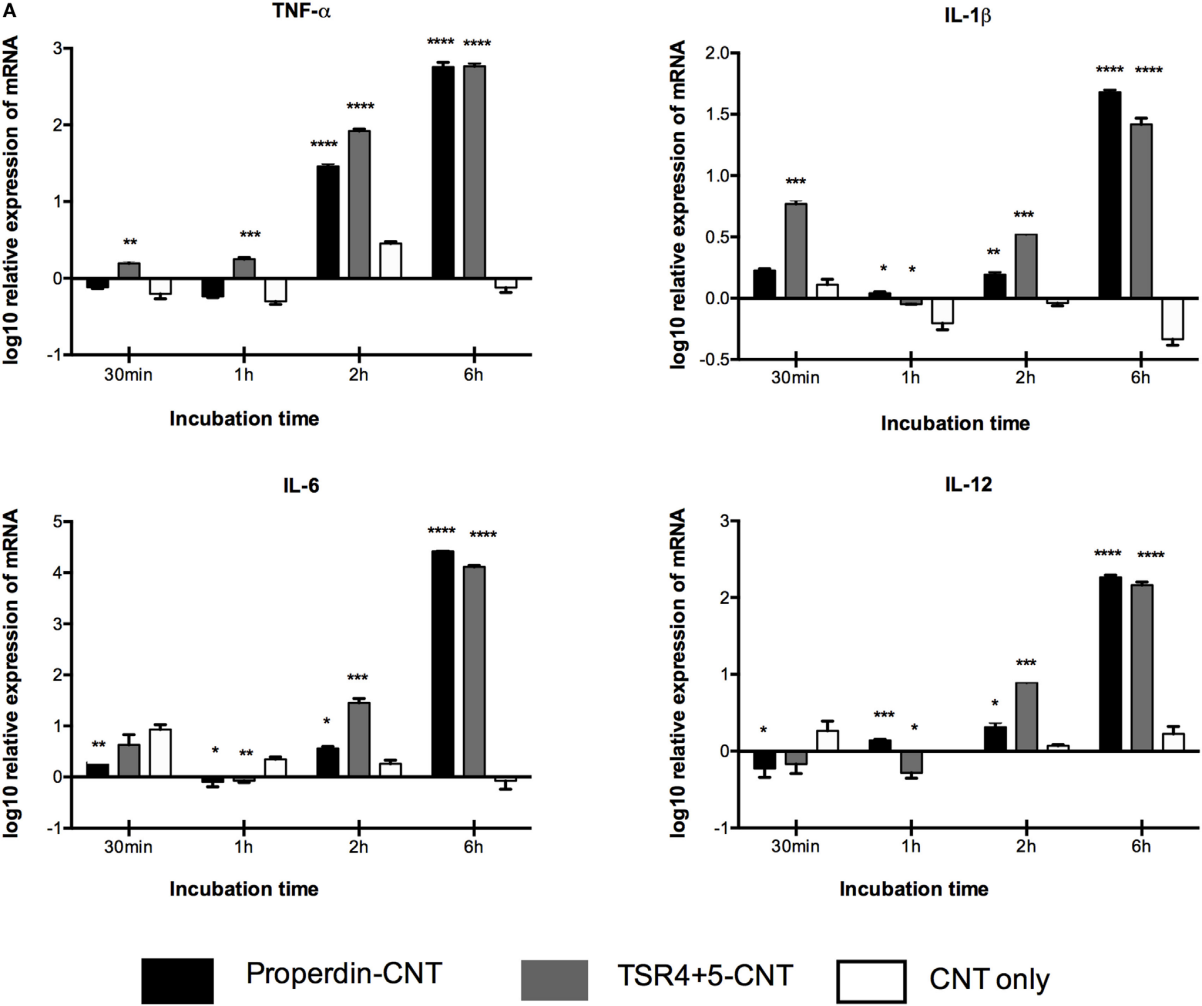

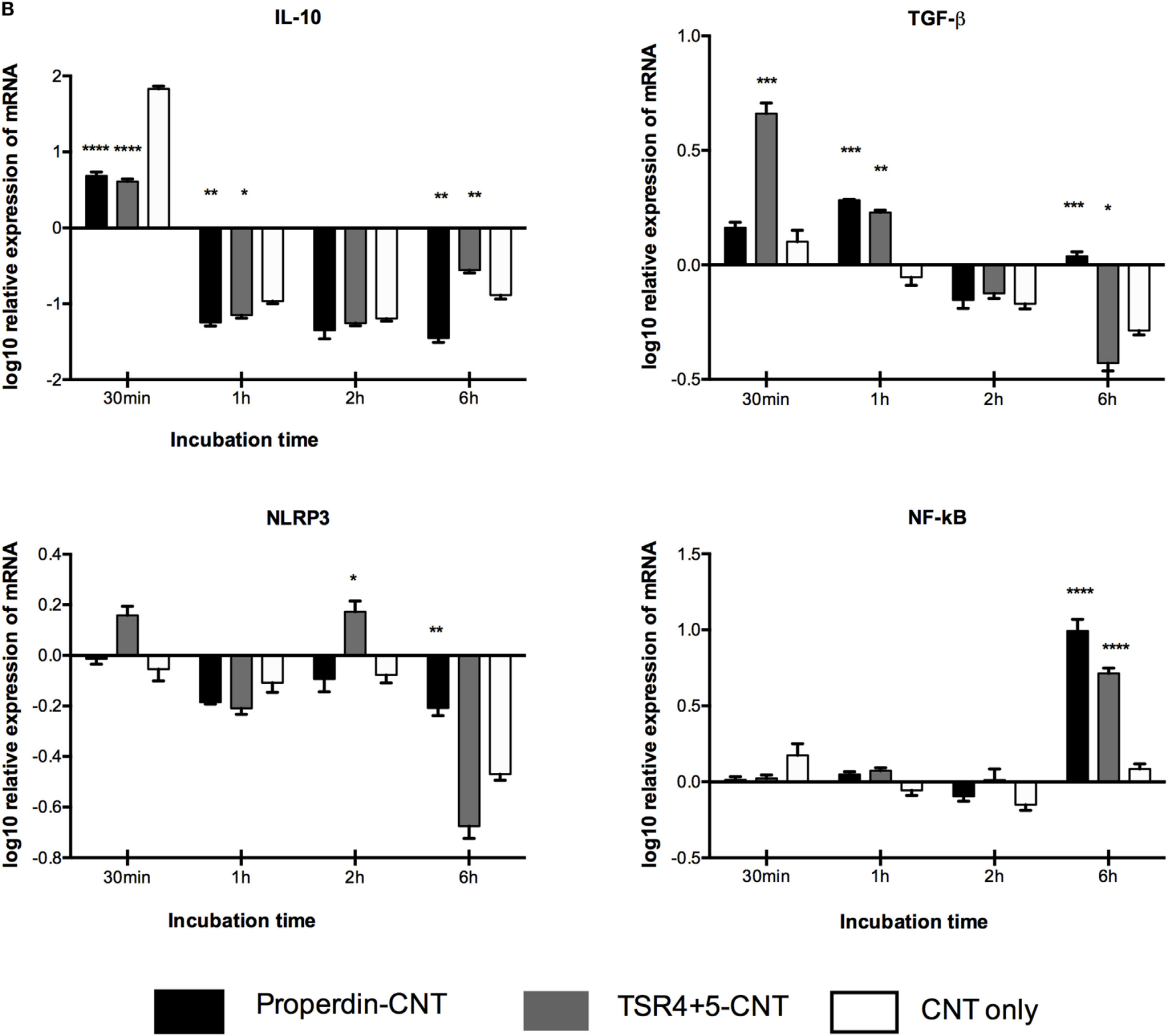
Transcriptional upregulation of pro-inflammatory cytokines by properdin or thrombospondin type I repeat (TSR)4 + 5-coated carboxymethyl cellulose-coated carbon nanotubes (CMC-CNTs). For the measurement of mRNA expression of **(A)** pro- and **(B)** anti-inflammatory target genes, THP-1 cells were incubated with coated and uncoated CMC-CNTs for 30 min, 1 h, 2 h, and 6 h (*X*-axis). The expression of cytokines was measured using real-time qPCR, and the data were normalized using as endogenous control; 18S rRNA gene expression assays were conducted in triplicate. Error bars represent ±SEM. A multiple *t*-test using Holm–Sidak method was performed to determine significance differences in expression between each uncoated and protein-coated carbon nanotubes (CNTs) of different time points. All these comparisons were significant: *P < 0.05, **P ≤ 0.01, ***P ≤ 0.001, and ****P ≤ 0.0001.

Having found properdin acting as an opsonin for CNTs, we next examined the pro- and anti-inflammatory cytokine response by THP-1 cells *via* qPCR analysis. TNF-α, IL-1β, IL-6, and IL-12 mRNA expression were significantly upregulated at 6 h by properdin-CMC-CNT and TSR4+5, and CMC-CNTs from 30 min onward (Figure 6A). In contrast, IL-10 and TGF-β were initially upregulated at 30 min, but decreased by 6 h, suggesting that the anti-inflammatory response was dampened (Figure 6B). Consistent with the upregulation of TNF-α, NF-κB was also upregulated by 6 h, when CMC-CNTs were coated with properdin or TSR4+5. However, the NLRP3 mRNA expression was not significant, suggesting that the activation of NLRP3 inflammasome was weak.

### Multiplex Array Analysis Revealed Dramatic Upregulation of Pro-inflammatory Cytokines/Chemokines by THP-1 Cells When Challenged with CMC-CNTs Coated with Properdin or TSR4+5

**Figure 7:**
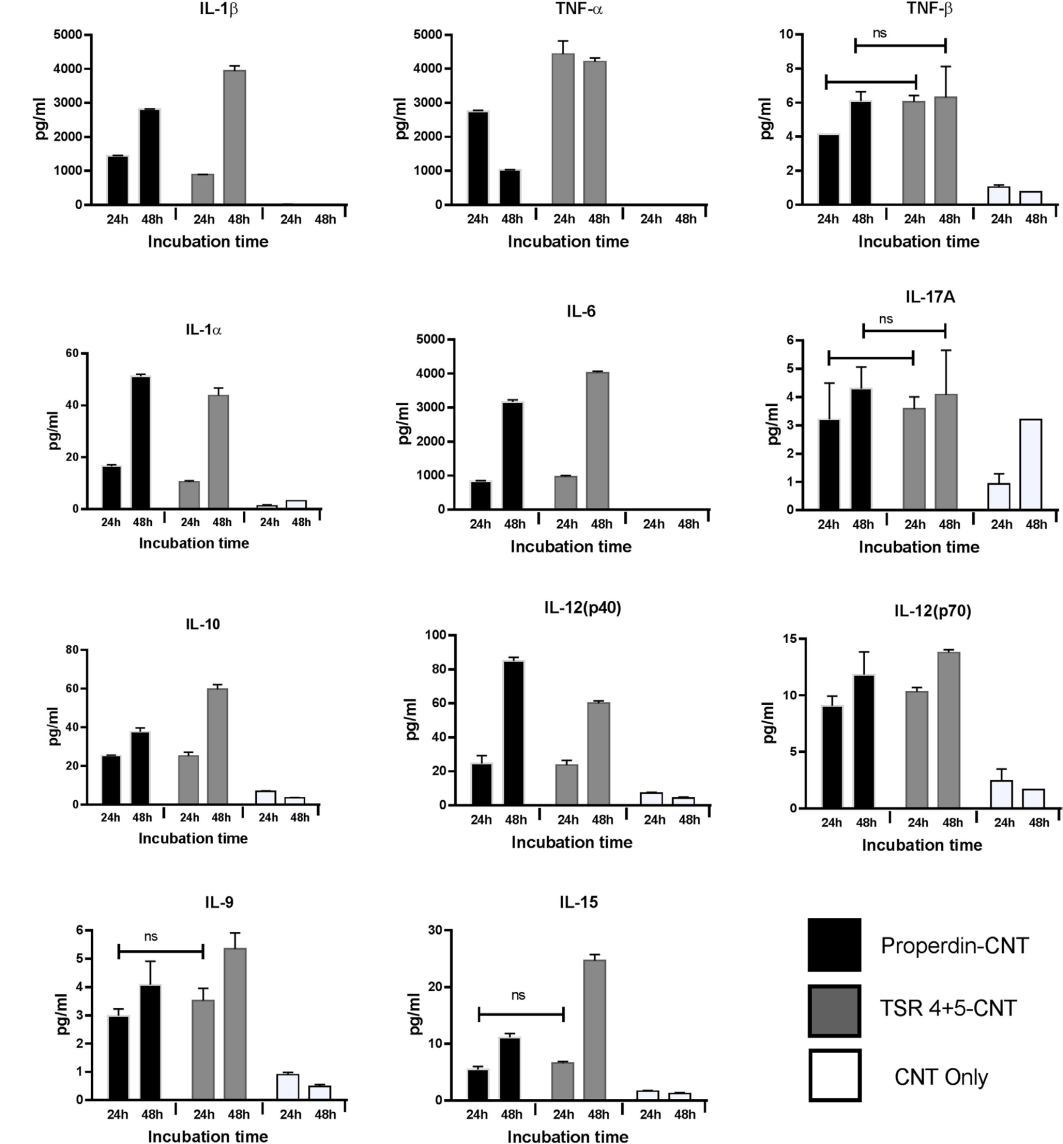

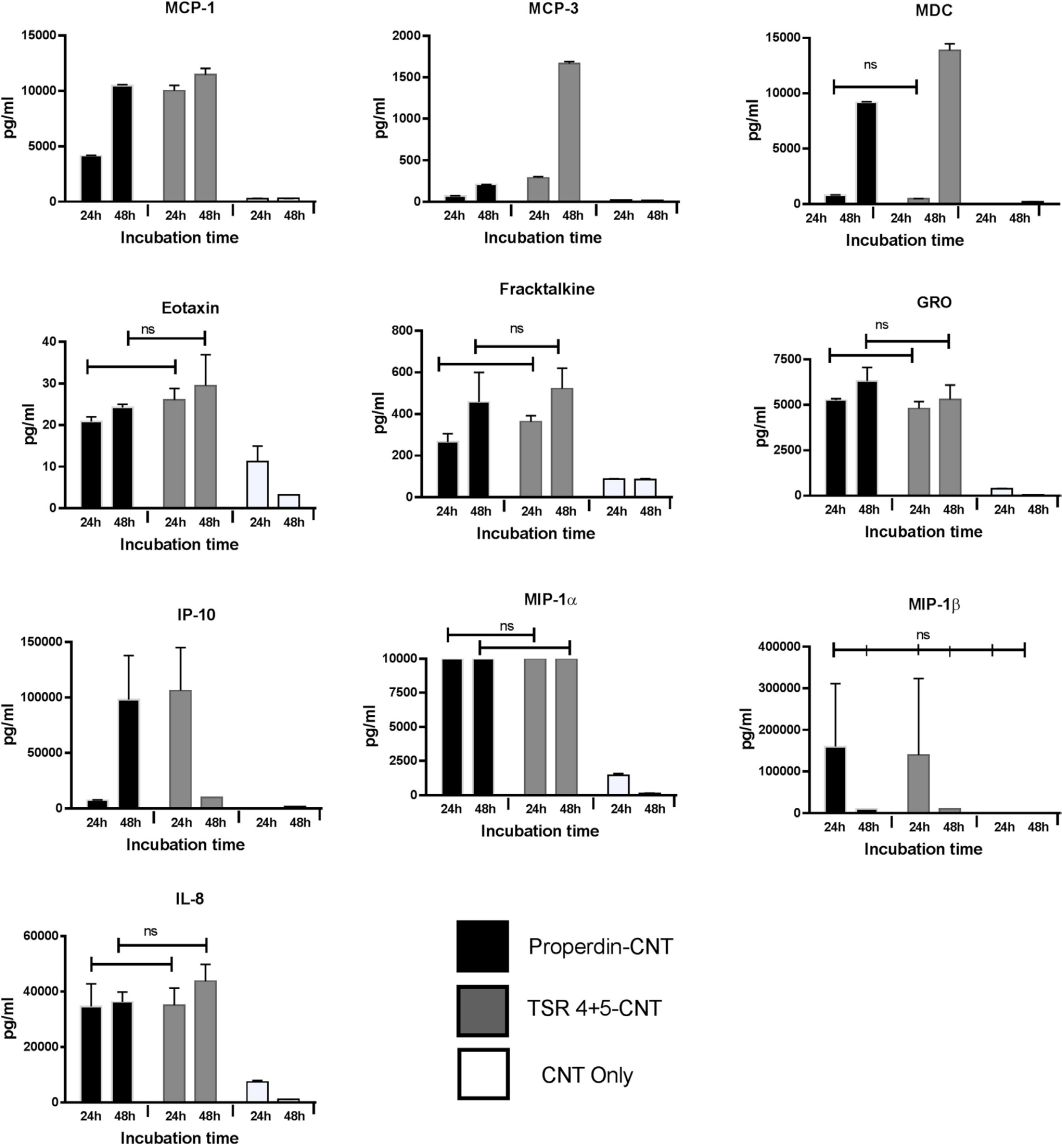

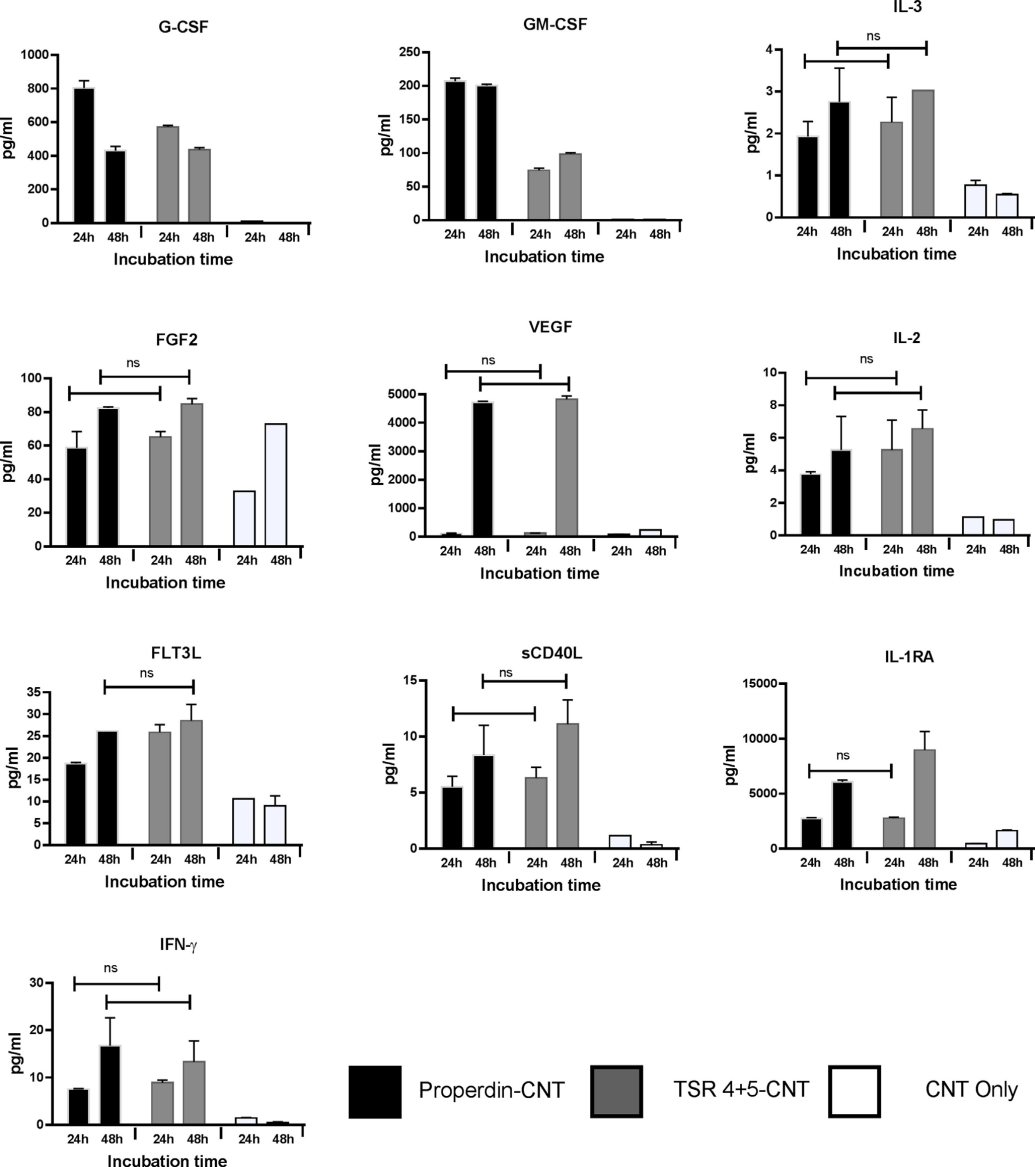
Properdin or thrombospondin type I repeat (TSR)4 + 5-coated carboxymethyl cellulose-coated carbon nanotubes (CMC-CNTs) induce secretion of pro-inflammatory cytokines and chemokines by THP-1 cells. Protein-coated carbon nanotubes (CNTs) were incubated with THP-1 cells for 30 min, 1 h, 2 h, 6 h, 12 h, 24 h, and 48 h. Cells from early time points (30 min, 1 h, 2 h, and 6 h) were used for quantitative expression of different cytokines. Supernatant from late time points (24 and 48 h) (*X*-axis) were used for the measurement of the levels of cytokines (IL-6, IL-10, IL12p40, IL12p70, IL-1α, IL-1β, TNF-α, IL-15, IL-17A, IL-9, and TNF-β), chemokines (MCP-3, MDC, eotaxin, fractalkine, GRO, IL-8, IP-10, MCP-1, MIP-1α, and MIP-1β), growth factors (IL-2, FGF-2, G-CSF, GM-CSF, IL-3, and VEGF), and related ligands and receptors (IFN-γ, FLT-3L, IL-1RA, and sCD40L) by using a commercially available MagPix Milliplex kit (EMD Millipore). A multiple *t*-test using Holm–Sidak method was performed to determine significance differences in expression between expression between properdin or TSR4+5-coated and uncoated nanoparticles of different time points. All these comparisons were significant (*P* < 0.05), except where shown [not significant (ns), *P* > 0.05].

Multiplex array analysis using supernatants that were collected at 24 and 48 h time points from the phagocytosis assay revealed a dramatic increase in the levels of pro-inflammatory cytokines (IL-6, IL-12p40, IL-12p70, IL-1α, IL-1β, TNF-α, IL-13, IL-15, and IL-9) for properdin-CMC-CNT or TSR4+5-CMC-CNT. Properdin and TSR4+5-coated CMC-CNTs also enhanced chemoattractants such as IL-8, I-TAC, MIG, and MCP-1 (Figure 7). A number of anti-inflammatory cytokines, chemokines, growth factors, and immune ligands were also differentially upregulated by protein-coated CNTs (Figure 7).

### Properdin or TSR4+5-Coated CMC-CNTs Induced Nuclear Translocation of NF-κB in THP-1 Cells

**Figure 8:**
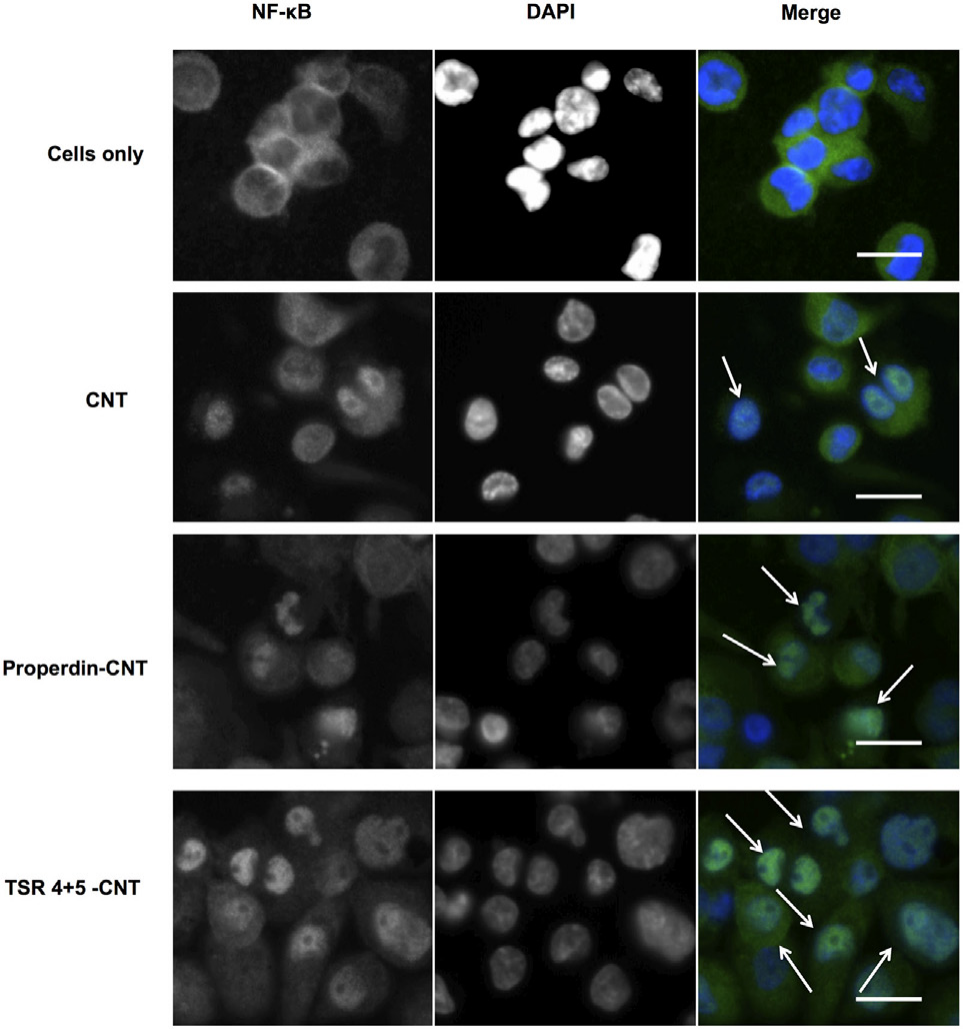
Carboxymethyl cellulose-coated carbon nanotubes (CMC-CNTs) coated with properdin or thrombospondin type I repeat (TSR) 4 + 5 cause translocation of transcription factor NF-κB from cytoplasm to nucleus of THP-1 cells (arrows in merged images). Differentiated THP-1 cells were treated with properdin-coated or TSR4+5-coated CMC-CNTs for 2 h. Cells were washed, fixed, permeabilized, and incubated with rabbit anti-NF-κB p65 antibodies, followed by secondary Alexa Fluor 488-goat anti-rabbit antibody (Green). The nucleus was stained with Hoechst 33342 (Blue). Scale bar: 20 µm. Hoechst 33342 was used to stain the nucleus instead of DAPI.

THP-1 cells were used to assess the translocation of NF-κB following challenge with Properdin-CMC-CNT or TSR4+5-CMC-CNT, using fluorescent staining. The transcription factor, NF-κB, regulates the signaling pathway of many pro-inflammatory cytokines when exposed to external stimuli. Properdin-CMC-CNT or TSR4+5-CMC-CNT incubated with THP-1 cells at 2 h were fluorescently stained with an antibody against the p65 subunit of NF-κB (green) (Figure 8). The merged image shows induction of translocation of NF-κB to the nucleus (blue), which is significantly enhanced by properdin-CMC-CNT and TSR4+5-CMC-CNT, compared to CMC-CNT alone (Figure 8). Properdin and TSR4+5-coated CMC-CNTs also induced an upregulation of NF-κB mRNA levels at 360 min by THP-1 cells (Figure 6B). This is consistent with the nuclear localization of NF-κB (Figure 7). This reflects on the upregulation of the pro-inflammatory response by TNF-α, IL-1β, IL-2, and IL-6 (Figures 6 and 7).

## Discussion

Previous reports have established an important role for the classical pathway in the recognition and phagocytic clearance of functionally derivatized CNTs (6, 8). CNTs, pristine and derivatized, appeared to offer a molecular charge pattern for C1q to bind and activate the classical pathway. On its own, C1q appeared to bind CNTs *via* its globular head (gC1q) domain, enhanced uptake of CNTs by macrophages, and upregulated the pro-inflammatory immune response (8). Although complement factor H also bound to derivatized CNTs, it did not enhance uptake while exerting an anti-inflammatory effect. CNTs, coated with recombinant forms of globular head modules corresponding to the *C*-terminal ends of the A, B, and C chains of C1q [ghA, ghB, and ghC, respectively (39)], were able to inhibit classical pathway activation *via* the nanoparticle surface. In addition, they also enhanced uptake of CNTs leading to considerable downregulation of a pro-inflammatory response. Thus, nanoparticles precoated with recombinant globular heads have been proposed to be a good strategy to avoid quick clearance of nanotherapeutics by phagocytes due to complement deposition with concomitant suppression of pro-inflammatory cytokine/chemokine response (8). Here, we have examined whether properdin can interact with nanoparticles, thus potentially acting as a pattern recognition innate immune molecule, similar to C1q and factor H, and modulate CNT handling by macrophages.

Pluronic-corona nanoparticles stimulate complement activation *via* the alternative pathway. The hydroxyl-containing nanoparticles are strong activators of C3, and the core thiols enhance the release of C3a (40, 41). Fe MWNTs coated with CMC, RNA or PL-PEG-NH_2_ activate both the classical (predominantly) and alternative pathways (6). The consumption of C3 and C5 on the CNT surface suggests that there would be binding sites for C4b and C3b. C3b and C4b bind to nucleophilic groups, such as OH, and NH_2_ groups; however, C3b does not form covalent adducts with high-molecular-weight proteins when bound to CNT. Thus, it may bind *via* hydrophobic interactions with CNTs or covalently to unesterified cholesterol in adsorbed HDL. Serum opsonization or complement deposition enhanced the uptake of CNTs by macrophages compared to non-opsonized CNTs, leading to an anti-inflammatory cytokine response (6).

Properdin is a highly positively charged molecule at neutral pH, with an isoelectric point of >9.5 (21). It binds to several self and non-self-target ligands such as Zymosan (42), rabbit erythrocytes, *N. gonorrhoeae* (32), T cells (27), human proximal tubular epithelial cells (43), and cartilage oligomeric matrix protein (44). Properdin engagement with surface-bound C3b recruits more C3b to form the C3 convertase complex, C3bBb. Properdin has also been proposed to act as a pattern recognition molecule for initiating alternative pathway other than just stabilizing the already formed C3bBb convertase (14). Properdin can directly interact with target ligands such as GAGs (28), including heparin (45), heparan sulfate (27, 46), dextran sulfate, fucoidan (47), and chondroitin sulfate (27). Properdin binding to activated platelets appears to occur *via* surface GAGs; when surface GAGs are removed, there is reduction in properdin binding to activated platelets (48). Properdin can bind DNA on late apoptotic and necrotic cells (42) and also bacterial LPS (33). Properdin’s direct interaction with cell surface molecules may indicate it is a selective pattern recognition molecule. Thus, properdin may be a key innate immune molecule that can bind to a wide range of nanotherapeutics.

Nanoparticles are hydrophobic but are made biocompatible by coating with CMC, which is negatively charged, making the particles water soluble, less toxic, and more biodegradable and biotolerable (49). Many cell surface molecules identified so far that interact directly with properdin are negatively charged. Properdin and TSR4+5 were able to bind CNTs (CMC-CNT and Ox-CNT) directly and stably (Figure 3), in a conformation that retained their biological activity (Figure 4). We next assessed the uptake of properdin-CMC-CNT and TSR4+5-CMC-CNT by THP-1 macrophages (Figure 5). Properdin, but not TSR4+5, was able to enhance the phagocytosis of nanoparticles considerably. It is likely that the properdin-mediated enhancement of phagocytosis requires additional TSR modules or multimers of properdin. This may be due to the requirement for additional receptors or more widespread engagement of receptors binding to whole properdin. Although uptake of nanoparticles was exclusive to properdin, TSR4+5 was also able to trigger the pro-inflammatory cytokine response similar to properdin. This may indicate that additional receptors need to be engaged for phagocytic uptake, which are not necessarily required for the cytokine response. CMC-CNTs on their own were not able to produce a significant signal for these cytokines; however, IL-10 mRNA was upregulated at 30 min, which may be dampening the activation of macrophages, thus explaining lesser uptake of uncoated CMC-CNTs alone. TNF-α, IL-1β, and IL-6 transcripts were dramatically upregulated at 6 h, suggesting that properdin or TSR4+5 coated CMC-CNTs may alter the immune response. Once the early response of nanoparticles was revealed by qPCR, we further analyzed the secreted cytokine levels at 24 and 48 h following THP-1-CMC-CNT interaction, using multiplex array analysis (Figure 7). Interestingly, a dramatic upregulation of pro-inflammatory cytokine response was observed consistent with the early mRNA response for TNF-α, IL-1β, and IL-6 (Figure 6), which are potent inducers of GM-CSF (50). GM-CSF was dramatically upregulated by Properdin-CMC-CNT as well as TSR4+5-CMC-CNT. GM-CSF is produced by macrophages in response to immune stimuli, which can recruit neutrophils and lymphocytes. IL-1α and IL-1β (Figure 7) induce an inflammatory pathway initiated *via* Myd88 activation and triggered by NF-κB transcription of inflammatory genes. In addition, IL-8, a chemoattractant for neutrophils, may induce local production of properdin by neutrophils and enhance activation of the alternative pathway (30).

Although nanoparticles are considered highly promising drug delivery platforms in a variety of disease conditions, their systemic administration into the human body and their intended target tissue can be affected by innate and adaptive immune components. It has been shown in many studies that CNTs potently activate complement. Complement deposition leads to enhanced particle uptake by complement receptor-bearing macrophages and B cells (9). Subsequently, it was found that complement deposition on CNTs was in fact advantageous due to suppression of the pro-inflammatory response and upregulation of anti-inflammatory cytokine production (9). Functionally derivatized CNTs (CMC-CNT and RNA-CNT) activated complement and became coated with complement proteins when treated with serum, while gold nanowires of similar size (51) were found to be poor activators of complement, while mounting a robust pro-inflammatory response, in contrast to complement-activating nanoparticles. We have shown here that properdin can act as an opsonin for nanoparticles without involving complement recruitment and activation and enhance their uptake and clearance by a macrophage cell line. It is possible that THP-1 cells synthesize a sufficient quantity of complement proteins (e.g., C3) to contribute to this apparent opsonization. The World Protein Atlas^2^ reports that Properdin RNA and trace C3 RNA is found in these cells. However, Takizawa et al. found that the addition of serum as a complement source was necessary to observe phagocytosis of apoptotic cells by activated THP-1 cells (52). In addition, we have shown complement deposition on CNTs invariably modulates an otherwise pro-inflammatory response toward anti-inflammatory immune response dominated by IL-10 (9). Interesting, pulmonary surfactant protein SP-D can also opsonise CNTs and induce a potent pro-inflammatory response by macrophages. However, SP-D bound CNTs, when treated with serum, continued to activate complement, suggesting that SP-D binding site on the CNTs is distinct from that required for complement deposition. Even more interestingly, complement deposition on SP-D-bound CNTs downregulated the pro-inflammatory cytokine and chemokine production; instead, the immune response by macrophages became anti-inflammatory as revealed by multiplex array analysis and NF-kB nuclear translocation assay (53). These points appear to suggest complement-independent effects of properdin when bound to CNTs. It is worthwhile to clarify here that we are examining here the functions of properdin as a PRR, in a context where local synthesis of properdin may be intended for its non-complement functions, where all complement components may not be present for the alternative pathway activation.

Another important data that merit discussion here, which is not the main thrust of this study, is that TSR4+5-bound CNTs dampened the alternative pathway activation *in vitro*. The recombinant TSR4+5 has recently been shown to compete with properdin in binding to C3b (and other ligands) and inhibit the alternative pathway in solution phase (26). In this study, TSR4+5 bound to the CNTs and inhibited the alternative pathway activation on the surface of the bound nanoparticles, suggesting that the nanoparticles may be exploited to present a potential complement inhibitor as an arrayed drug delivery platform (Figure 4). These results are of pathophysiological significance because properdin deficiency has been linked with a range of bacterial infections especially *Neisseria* (54). The properdin gene-deficient mice have been found to be susceptible to bacterial infections (38). Properdin-CNTs used as a platform for drug delivery may mediate a protective role in disease mice models deficient in properdin. This will pave the way for further testing of its prophylactic and therapeutic values in murine models where properdin deficiency renders the mice susceptible to a range of infections.

Inhibition of complement alternative pathway has been previously reported to be beneficial in various pathological conditions. For example, mAb 1379, an anti-mouse factor B antibody, has been shown to provide protection against anti-phospholipid antibody-induced complement activation and fetal loss (55). Anti-C5 monoclonal antibody eculizumab is used as a therapeutic for paroxysmal nocturnal hemoglobinuria. We have reported in this study that TSR4+5:CMC-CNT are able to inhibit the alternative pathway by inhibiting consumption of complement on nanoparticles, which may have potential implications on therapeutic drug delivery in conditions with alternative pathway related diseases. Recently, a significant contribution of complement proteins to the tumor microenvironment has become a focus of intense research. Tumor cells thrive on an immunosuppressed microenvironment. To this effect, conditioned medium from melanoma B16F10 tumor cells has been shown to prime M1 macrophages, characterized by upregulation of iNOS, IL-1β, TNF-α, IL-12, IL-23, CXCL-9, and CXCL-10 (31). Thus, the tumor microenvironment could potentially be modulated by properdin-CNTs.

## Author Contributions

LK and BP carried out crucial experiments, plotted the data, and wrote the first draft. AK, GS, LJ, EF, SA, CS, and RS provided crucial reagents and expertise. UK led the work, designed experiments, analyzed the data, and finalized the manuscript.

## Conflict of Interest Statement

The authors declare that the research was conducted in the absence of any commercial or financial relationships that could be construed as a potential conflict of interest. The reviewer MD and handling editor declared their shared affiliation.

## Footnotes

^1^http://blast.ncbi.nlm.nih.gov/Blast.cgi.

^2^http://www.proteinatlas.org.
